# Mevalonate Kinase-Associated Diseases: Hunting for Phenotype–Genotype Correlation

**DOI:** 10.3390/jcm10081552

**Published:** 2021-04-07

**Authors:** Guilaine Boursier, Cécile Rittore, Florian Milhavet, Laurence Cuisset, Isabelle Touitou

**Affiliations:** 1 Department of Medical Genetics, Rare Diseases and Personalized Medicine, Rare and Autoinflammatory Diseases Unit, CHU, 34295 Montpellier, France; g-boursier@chu-montpellier.fr (G.B.); cecile.rittore@inserm.fr (C.R.); florian.milhavet@inserm.fr (F.M.); 2 IRMB, University of Montpellier, INSERM, 34295 Montpellier, France; 3 Genetic and Molecular Biology Laboratory, Cochin Hospital, 75014 Paris, France; laurence.cuisset@aphp.fr

**Keywords:** autoinflammatory diseases, mevalonate kinase deficiency, mevalonic aciduria, hyper-IgD syndrome, porokeratosis, phenotype–genotype correlation

## Abstract

Mevalonate kinase-associated diseases (MKAD) are caused by pathogenic mutations in the mevalonate kinase gene (*MVK*) and encompass several phenotypically different rare and hereditary autoinflammatory conditions. The most serious is a recessive systemic metabolic disease called mevalonic aciduria, and the most recently recognized is disseminated superficial actinic porokeratosis, a dominant disease limited to the skin. To evaluate a possible correlation between genotypes and (1) the different MKAD clinical subtypes or (2) the occurrence of severe manifestations, data were reviewed for all patients with *MVK* variants described in the literature (N = 346), as well as those referred to our center (N = 51). The genotypes including p.(Val377Ile) (homozygous or compound heterozygous) were more frequent in mild systemic forms but were also sometimes encountered with severe disease. We confirmed that amyloidosis was more prevalent in patients compound heterozygous for p.(Ile268Thr) and p.(Val377Ile) than in others and revealed new associations. Patients homozygous for p.(Leu264Phe), p.(Ala334Thr) or compound heterozygous for p.(His20Pro) and p.(Ala334Thr) had increased risk of severe neurological or ocular symptoms. All patients homozygous for p.(Leu264Phe) had a cataract. The variants associated with porokeratosis were relatively specific and more frequently caused a frameshift than in patients with other clinical forms (26% vs. 6%). We provide practical recommendations focusing on phenotype–genotype correlation in MKAD that could be helpful for prophylactic management.

## 1. Introduction

Monogenic autoinflammatory diseases are a group of rare and heterogeneous conditions that display abnormal regulation of innate immunity resulting from one (dominant transmission) or two (recessive transmission) pathogenic variants in a single gene [1]. Among them are diseases associated with the mevalonate kinase gene (*MVK*). Mevalonate kinase (MK, enzyme commission EC 2.7.1.36) is a key enzyme in the biosynthesis of cholesterol and isoprenoids. Defective protein prenylation underlies the inflammation seen in mevalonate kinase-associated diseases (MKAD) [2,3,4]. These include several very different phenotypic subtypes with either systemic [5] or localized [6] heterogeneous manifestations. Different modes of inheritance have also emerged [7,8].

The systemic subtypes are recessive diseases named mevalonate kinase deficiency (MKD). MKD encompasses a continuum of conditions ranging from a serious metabolic disease called mevalonic aciduria (MA, OMIM—Online Mendelian Inheritance in Men, 610377) [2,9] to a milder disease coined “hyper IgD (Immunoglobulin D) syndrome with periodic fever” (HIDS, OMIM 260920) because high amounts of IgD were found in the serum of most patients [7,10]. However, this IgD measurement should be abandoned in daily practice because elevated IgD level is neither specific nor constant [11] and does not correlate with disease severity, mevalonate kinase enzyme activity, or genotype [12]. MKD symptoms occur in early childhood and are associated with elevation of nonspecific biological markers of inflammation (high level of C-reactive protein) and a biochemical signature (mevalonic aciduria during febrile episodes and decreased MK enzyme activity in cultured fibroblasts or leukocytes) [13]. In the milder form, typical disease is characterized by recurrent fever lasting 3 to 7 days associated with abdominal pain, arthralgia, adenopathy, and increased susceptibility to infections. Skin manifestations are present in more than two-thirds of patients. A maculopapular rash is the most frequent cutaneous manifestation, but urticarial rash, purpura, erythema nodosum, and oral aphthous ulcers have also been reported [14]. In MA, the inflammatory symptoms are often hidden by severe neurological manifestations such as cerebellar ataxia, seizures, and/or mental, motor, and growth retardation [2,15]. Atypical forms include nonsyndromic retinitis pigmentosa (RP) [16,17,18,19], prominent liver [20] or cardiorespiratory disease [21,22], inflammatory bowel disease (IBD) [23], or amyloid A amyloidosis [24,25,26,27].

Disseminated superficial actinic porokeratosis (DSAP) is a MKAD subtype localized to the skin and characterized by epidermal keratinization [28]. Both dominant germline and somatic *MVK* mutations have been described in these patients [29,30].

Given the wide spectrum of phenotypes associated with *MVK* and the diagnostic delay that may result, we investigated possible phenotype–genotype correlation in MKAD to determine whether prophylactic care could be improved on the basis of the mutations carried by the patient. We first reviewed the literature, which showed that little is known about this issue. This observation prompted us to design a study on the largest possible series of patients with all MKAD subtypes to try to identify clearer genotype–phenotype correlations after the integration of newer patients.

## 2. Experimental Section

### 2.1. Data Collection

**Figure 1:**
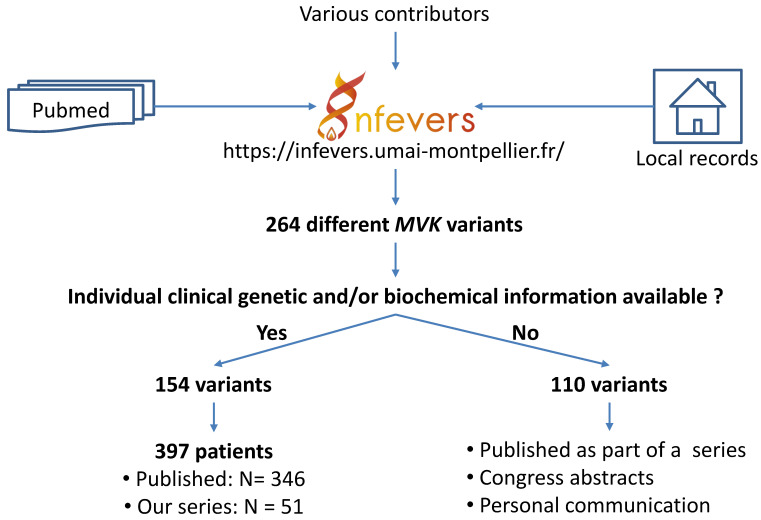
Steps leading to the identification of patients with sequence variants in the *MVK* gene.

Raw data were extracted for all published cases (N = 346) with available clinical and genetic information collected from the text, tables or supplemental material, as well as from the records of our unpublished cases (N = 51) (Figure 1).

Three resources were queried for this study (last search 12 March 2020): (1) references extracted from PubMed with the following string of keywords: (*MVK* or mevalonate kinase or mevalonic aciduria or hyperIgD syndrome or HIDS), (2) records of patients referred to our laboratory for genetic analysis, and (3) Infevers, the registry of autoinflammatory mutations, which was updated with the two other resources if necessary. Care was taken to exclude potential patients reported more than once by matching, where available, years of birth, disease onset or diagnosis, as well as gender, registry number, genotype, phenotype, references, and authors. For systemic MKD, we selected patients with two disease-causing *MVK* variants, and also retained data from early publications for 9 patients with only one pathogenic variant detected, but whose diagnosis was confirmed by decreased enzyme activity or elevated mevalonic aciduria level.

### 2.2. Statistical Analysis

Comparison between subtypes involved two-tailed Fisher exact test (https://biostatgv.sentiweb.fr/?module=tests/fisher (accessed on 2 September 2020)), with Bonferroni correction for multiple comparisons.

## 3. Results

### 3.1. Review of the Literature

Few series have attempted to formally establish a correlation between genotype and the patient’s clinical or biochemical phenotype.

#### 3.1.1. Genotype vs. Clinical Phenotype

Shortly after the gene discovery, Hinson et al. in 1997 identified the first *MVK* pathogenic variant p.(Ala334Thr), responsible for MKD-MA [31]. This variant was later mostly found in severe or atypical MKD.

Simon et al. and Houten et al., in the early 2000s, evaluated the distribution of *MVK* variants in MKD. They discovered that p.(Val377Ile), the most frequent variant likely because it is derived from a founder effect, was mainly reported in MKD-HIDS [32,33]. Two other common pathogenic variants, p.(Ile268Thr) and p.(His20Pro), were seen in all MKD subtypes. The authors identified patients with phenotypic overlaps, thus advocating for a phenotypic continuum [15]. They suggested that patients with two mild variants (e.g., p.(Val377Ile)) would have MKD-HIDS, patients with two severe mutations (e.g., p.(Ala334Thr)) would have MKD-MA, and patients with two intermediate variants (e.g., p.(His20Pro) or p.(Ile268Thr)) would have an intermediate phenotype.

Cuisset et al., in 2001, compared the phenotype of 14 patients who were compound heterozygotes for p.(Val377Ile) to 5 patients who did not carry this variant [34]. However, the groups did not differ in frequency or severity of febrile attacks or symptomatology during attacks. This finding probably reflects a lack of power related to the sample size available at the time.

Ter Haar et al., in 2016, examined 13 clinical features in four genotypically different groups of MKD patients: (1) homozygous for p.(Val377Ile), (2) compound heterozygous for p.(Val377Ile) and p.(Ile268Thr), (3) compound heterozygous for the p.(Val377Ile) variant and a second variant other than p.(Ile268Thr), and (4) without the p.(Val377Ile) variant [35]. A chronic disease and severe gastrointestinal and musculoskeletal impairment prevailed in patients without p.(Val377Ile), whereas those with p.(Val377Ile) and p.(Ile268Thr) were more likely to have amyloidosis.

#### 3.1.2. Genotype vs. Biochemical Phenotype

Mandey et al., in 2006, analyzed the *MVK* gene in 57 patients with MKD and observed rather good correlation between genotype and both clinical and biochemical features [36]. Nevertheless, the authors found that measuring residual MK enzyme activity seemed more informative than predicting the pathogenic effect that could result from the altered protein structure induced by certain sequence variants.

Conversely, in 2016, Jeyaratnam et al. suggested that detection of pathogenic urinary levels of mevalonic acid should not be mandatory before genetic testing [37]. Indeed, the authors ruled out a direct and reliable relation between genotype and biochemical measurements. For example, a patient homozygous for p.(Val377Ile) with defective leucocyte enzyme activity (2–3% compared to controls) showed normal aciduria excretion, although urine was correctly collected during a fever episode. Indeed, practical difficulties in obtaining urine mevalonic acid in some clinical practices, as well as lab-to-lab variability, may affect the reliability of this test. In another study, a patient with a homozygous p.(Val377Ile) genotype, typical MKD symptoms, and high urinary excretion of mevalonic acid showed normal enzyme activity in both fibroblasts and leukocytes [35].

#### 3.1.3. Genetic Predictions

Browne et al. developed an in silico approach to evaluate the effect of 67 *MVK* variants on MK structure, stability, and function [38]. Physicochemical analysis of all variants highlighted a tendency to predict decreasing structural stability with increasing severity of the disease-associated variants. The authors suggested that their method could be used as a basis for initial severity predictions when new *MVK* variants are discovered, but concluded that experimental testing will be necessary to confirm these predictions.

A consortium of genetic experts used the recommendations of the American College of Medical Genetics supplemented with their own unpublished data to classify and establish a pathogenicity score for all *MVK* variants known at that time [39]. A classification was considered validated if ≥75% of the experts reached consistent votes. A provisional classification was assigned if between >50% and 75% of experts reached consistent votes. Variants that did not fulfil those criteria remained with the status “classification unsolved”. This score gives the probability that a given variant is pathogenic or not, but does not reflect the disease subtype (systemic or dermatological forms) or severity (mild or severe).

Table S1 lists these two different predictions.

### 3.2. Raw Data Mining

In an attempt to better understand the possible links between the patients’ genotype and the different MKD subtypes and clinical manifestations, we assembled data from published and unpublished cases (Table S2). For the sake of simplicity, the phenotype subtype was kept as published in the title or body of the manuscript. We identified 128 patients described with the generic term “MKD”, 100 MKD-HIDS patients, 74 PK patients, 56 patients with atypical features (RP, IBD, amyloidosis, prominent liver, cardiorespiratory disease), 38 MKD-MA patients, and one asymptomatic relative. All subtypes were consistent within the same family, except in two siblings homozygous for p.(Val377Ile): one sister had MKD-HIDS and the other had no symptoms [13,40]. The 397 patients studied in this work included 347 probands, 38 from multiplex families, and 309 simplex cases (Table S2), in whom we identified 154 (Table S1) of the 264 known *MVK* variants at the time of this study (https://infevers.umai-montpellier.fr/web/index.php (accessed on 12 March 2020) [41].

#### 3.2.1. Impact of the Variant Location on the MKAD Subtypes

**Figure 2:**
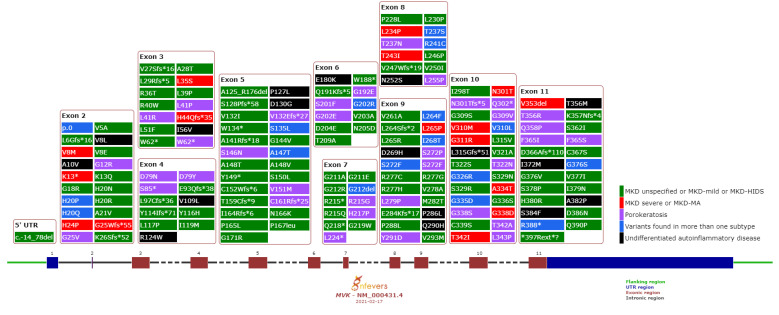
Sequence variants in the *MVK* gene (NM_000431.2). This schema has been generated from the “build your graph” module of Infevers (https://infevers.umai-montpellier.fr/web/graph.php?n=3&wid=1280&hei=720), (last search 12 March 2020). Benign and likely benign variants are not shown.

Although we did not expect that a single allele could determine the phenotypic subtype of a recessive disease, we looked if certain variants clustered in a specific region of the gene. In general, we did not detect any preferential localization according to the subtype (Figure 2). However, variants found in MA were clustered within two hotspots, lying between residues 8–44 and 234–353, in good agreement with previous observations [38].

#### 3.2.2. Impact of MVK Genotypes on MKAD Subtypes

**Figure 3:**
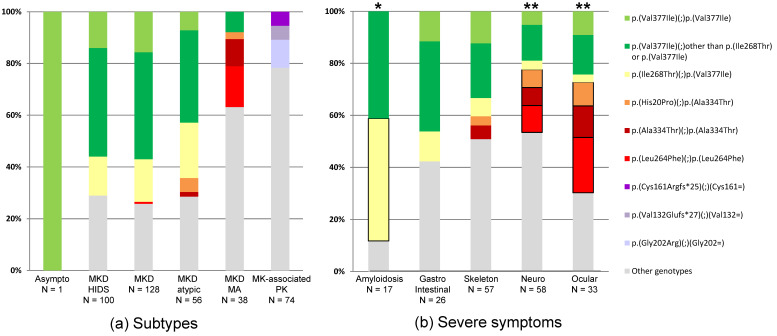
Distribution of the most frequent genotypes in mevalonate kinase-associated diseases patients. (**a**) According to mevalonate kinase-associated diseases subtypes. The mevalonate kinase-associated diseases (MKAD) subtypes are as reported: MKD: mevalonate kinase deficiency, systemic undefined; HIDS: hyper-IgD (Immunoglobin D) syndrome; MA: mevalonic aciduria; PK: porokeratosis. (**b**) According to the presence of severe symptoms defined as follows: gastrointestinal: adhesion, ascitis, bleeding, cholestasis, inflammatory bowel disease, and occlusion; skeleton: arthritis, bone sign, and dystonia; neurological: allodynia, ataxia, cerebral atrophy, language disorder, mental or motor retardation, paralysis, seizures, stroke, tremor, and meningitis; ocular: cataract, episcleritis, keratopathy, loss of vision, optic atrophy or neuritis, retinopathy, and uveitis. The boxed genotypes (alone for amyloidosis, or pooled for neurological and ocular symptoms) depict those significantly associated with severe manifestations within a given group of symptoms: * *p* < 0.001; ** *p* < 0.0001.

For the recessive MKD subtypes, we considered the cumulative effect of the two alleles. The two most frequent genotypes were p.(Val377Ile) compound heterozygous with p.(Ile268Thr) (12%) and p.(Val377Ile) homozygous (10%); both were observed in MKD, MKD-HIDS, and MKD-atypical but not in MKD-MA (Figure 3a).

In the dominantly inherited PK form, genetic specificities were observed. First, the three most common heterozygous variants, p.(Val132Glufs*27), p.(Gly140Argfs*47), and p.(Cys161Argfs*25), all caused frameshifts. The proportion of such variants was significantly higher in patients with PK than in those with systemic MKD forms (19/74, 26%, vs. 19/323, 6%; *p* < 10^−6^), whereas all other variant types were evenly distributed among the MKAD subtypes. Second, all variants associated with PK, with the exception of three affecting a glycine residue (p.Gly212del, p.Gly335Asp, and p.Gly376Ser), were not found in the other subtypes (Figure 3a and Table S2). These observations are still unexplained. These different mutations likely have a differential impact on the function and regulation of the protein in the cell.

#### 3.2.3. Impact of MVK Genotypes on Severe MKD Manifestations

The study next addressed whether certain severe symptoms could be associated with a specific genotype (Figure 3b), focusing on the best-documented and most discriminating damage items available [42].

Amyloidosis is rare in MKD. A recent review estimated its prevalence at about 6% [27], which is consistent with the 5% of MKD patients collected here with amyloidosis (N = 17/323). In total, the genotypes of 15 of the 17 patients included at least one p.(Val377Ile) variant, 8 being heterozygous with p.(Ile268Thr). The difference between these 8 patients and those with other genotypes was significant (*p* < 0.001), formally confirming a previous observation [35].

With respect to other clinical features, 26 (7%) patients had severe skeletal manifestations and 57 (14%) severe gastrointestinal manifestations. Overall, 57% and 60%, respectively, had at least one p.(Val377Ile) variant, but the difference with patients with other genotypes was not significant. No other specific risk genotypes were revealed. In all, 58 (15%) and 33 (8%) patients had severe ocular and severe neurological symptoms. When combined, the three genotypes found most frequently in these patients, p.(Leu264Phe) homozygosity, p.(Ala334Thr) homozygosity, and p.(His20Pro) compound heterozygous with p.(Ala334Thr), were strongly associated with these severe features as compared with all other genotypes (*p* < 0.0001). Specifically (Table S2), all 7 patients homozygous for p.(Leu264Phe) had a cataract, and 13/15 (87%) patients with cataract had a genotype comprising p.(Leu264Phe) or p.(Ala334Thr). Unexpectedly, three patients with neurological defects [13,40,43] and/or ocular defects [13,40,44] were homozygous for p.(Val377Ile).

#### 3.2.4. Impact of Non-MVK Variants on the Phenotype

Additional variants in other genes responsible for autoinflammatory disorders were sometimes revealed when genetic diagnosis was performed by sequencing gene panels (Table S2). The two most frequent variants, both classified as of uncertain significance, were p.(Glu148Gln) in *MEFV*, and p.(Arg121Gln), historically known as R92Q, in *TNFRSF1A* [41]. All subtypes except MKD-MA were found in patients with non-MKD variants. They were too few to draw any serious statistics. However, interestingly, a STAT1 (Signal Transducer And Activator Of Transcription 1) variant, p.(Arg241Gln), was identified in only one of two siblings homozygous for the p.(Val377Ile) genotype. One was asymptomatic and the other had a rather severe phenotype [13,40].

## 4. Discussion

Diseases associated with *MVK* pathogenic variants, herein jointly referred to as MKAD, are heterogeneous at all levels, clinical, biochemical, and genetic [2,45,46]. Indeed, MK is an essential, ubiquitously expressed enzyme, whose expression is also affected by the patient’s genetic and environmental background, for example, temperature [36]. In patients with MKD-HIDS, defective enzymatic activity varies widely, whereas very high concentrations of mevalonic acid are present constitutively in urine in patients with MKD-MA [34]. Munoz et al. recently proposed that MKD can be identified and distinguished from other autoinflammatory disorders by the defect in protein prenylation, and that this defect can be detected using an in vitro prenylation assay, which accurately detects the accumulation of unprenylated proteins in peripheral blood mononuclear cells [4]. However, it remains unclear how the severity of mutations in *MVK* correlates with loss of MK enzyme activity. This study addressed and raised several genetic questions related to phenotype–genotype correlations in these patients.

One issue concerns the possibility for the geneticist to accurately classify variants to predict phenotype from genotype and for the clinician to unambiguously classify patients into a specific clinical form of MKAD. The review of the literature has shown that although this classification could be useful for the clinician, the confinement of a variant to a unique prediction regardless of the patient’s genetic background, or a phenotype to a particular subtype is not always relevant for diagnosis and therefore genetic counselling. For example, variants with a mild prediction such as p.(Val377Ile) were also found in patients with severe clinical manifestations (Table S2 and Figure 3); some patients with MKD-HIDS actually have amyloidosis [27] or RP [15,16,17,18,19], both serious complications.

To answer this question in greater depth, we retrieved all possible cases from the literature and supplemented them with our center’s cases. Despite the limitations of such a study design (retrospective and incomplete collection of information to delineate the quality of clinical data), there were several benefits. First, the present series is the most exhaustive to date (nearly 400 patients). Second, its gateway was the genotype, regardless of patient phenotype, thus circumventing the risk of subjective inclusion or exclusion criteria. Third, the study considered the risk conferred by the genotype rather than individual alleles, a particularly relevant issue, at least for the recessive subtypes of MKAD.

Although this study did not replicate some preliminary results that emerged from the Eurofever cohort, i.e., difference between patients with genotypes not including p.(Val377Ile) versus all other groups for severe gastrointestinal and musculoskeletal symptoms, it strongly confirmed the different distribution of neurosensory defects between these two genotypic groups [35]. In addition, it revealed a significant association between severe neurological and ocular manifestations (particularly cataract) and genotypes including the two variants p.(Ala334Thr) and p.(Leu264Phe). This was expected but had never been formally demonstrated [15]. The study also clearly confirmed the association of amyloidosis with compound heterozygosity for p.(Ile268Thr) and (Val377Ile), an observation showing borderline significance in a smaller series [35]. Therefore, carrying p.(Val377Ile) does not prevent the occurrence of this renal complication. Finally, the compilation of patients with PK revealed a range of associated genotypes, most completely different from those involved in systemic forms of MKAD. The main messages from this work are summarized in Table 1.

A second related question concerns the biological and/or environmental determinants that cause one phenotypic subtype versus another. There is no doubt that genes other than the causative *MVK* affect disease manifestation. Recently, Carapito et al., with multi-OMICS analyses, identified a variant of STAT1 that was differentially expressed in two sisters homozygous for the p.(Val377Ile) genotype who had radically opposite (asymptomatic vs. strongly symptomatic) phenotypes, thus providing an initial clue to the low penetrance associated with this variant [47]. This finding most likely explains why p.(Val377Ile) homozygosity is not the most common genotype in MKAD patients with overt disease (Table S2). A constellation of functional polymorphisms is also probably involved in the phenotype. For example, a common genetic background has been postulated between MKD and early onset IBD [23] or Behçet’s disease [44]. The current review highlighted several patients who co-inherited variants in other genes (Table S2), but experimental approaches are needed to ascertain their possible role in MKAD. Finally, epigenetics and environmental factors are likely to play a role in MKAD. For example, DSAP lesions resulting from second-hit somatic mitotic recombination or point mutations with a UV signature were recently detected [29,48].

In conclusion, this work provides new and practical recommendations focusing on phenotype–genotype correlations in MKAD that could be helpful for prophylactic care of patients. The increased availability of routine exome or genome sequencing will further make it possible to take into account the cumulative effect of all *MVK* alleles inherited by the patient, as well as possible other modifier genes.

## Figures and Tables

**Table 1:** Mevalonate kinase-associated diseases take-home messages.

Target	Observation	Lesson
Clinical care	Measurement of IgD	To be dropped
	Mevalonic aciduria	Elevated, but anecdotally normal
	Amyloidosis	Rare but does exist as in any other hereditary recurrent fevers
Phenotype genotype correlation	In Silico prediction tools	Helpful, but no strict correlation
	“Mild” p.(Val3777Ile) compound heterozygous or homozygous state	Also found in severe MKD, although anecdotally
	p.(Leu264Phe) or p.(Ala334Thr) homozygosity	High risk of severe ocular or neurological manifestations
	p.(Leu264Phe) homozygosity	100% association with cataract (early ophthalmological test recommended?)
	MKAD-PK	Specific range of pathogenic variants (frameshifts). Poor overlap (glycines) with systemic forms. Second mutation to search in cutaneous lesions

MKD: Mevalonate kinase deficiency. PK: porokeratosis.

## Data Availability

All data are available in the Two Supplementary Tables.

## Supplementary Materials

The following are available online at https://www.mdpi.com/article/10.3390/jcm10081552/s1, Table S1: Distribution of the 154 different mevalonate kinase (*MVK*) genetic variants identified in probands with mevalonate kinase-associated diseases, Table S2: Detailed description of 397 patients with mevalonate kinase (*MVK*) genetic variants according to their phenotype.
